# Molecular replacements

**DOI:** 10.1107/S0907444913027352

**Published:** 2013-10-18

**Authors:** Charles Ballard, Pietro Roversi, Helen Walden

**Affiliations:** a Diamond Light Source Ltd, Harwell Science and Innovation Campus, Didcot OX11 0DE, UK; b Oxford University Biochemistry Department, South Parks Road, Oxford OX1 3QU, UK; c Cancer Research UK, London Research Institute, Lincoln’s Inn Fields Laboratories, 44 Lincoln’s Inn Fields, London WC2A 3LY, UK

**Keywords:** CCP4 Study Weekend 2013

## Abstract

An introduction to the proceedings of the CCP4 Study Weekend held at the East Midlands Conference Centre of the University of Nottingham, England, in January 2013.

The collection of articles in this issue forms the proceedings of the 2013 CCP4 Study Weekend *Molecular Replacements*, which took place on 3–5 January 2013 at the East Midlands Conference Centre of the University of Nottingham, England, UK. As the plural in the chosen title suggests, molecular replacement (MR) these days is relevant in many a field of structural science, well beyond the original task of placing a homologous model in a crystal’s unit cell based on its experimentally measured structure-factor amplitudes. In the papers gathered here, MR is cast in contexts as wide and varied as the history of structural science; protein fold and family structural coverage; protein structure modelling; electron microscopy of macromolecular complexes; structural virology; and of course crystallographic software development.

As you will see, the 2013 CCP4 Study Weekend has reminded us that MR has an undoubtedly glorious past, and its present is just as vital and full of interesting new developments: but it is the future of MR that looks brightest. Marco Punta’s contribution to this volume offers a glimpse of a time at which, once a few structural representatives are known for all known protein families, MR is likely to become the technique of choice to obtain initial phases for the majority of future macromolecular crystal structures. As if we needed to be reminded of the importance of those structures, the paper points out that there are a thousand or so protein families to which at least one human protein belongs, and for which some biological but no structural information is available. Keep up the good work, structural biologists! And may MR always come to your rescue.

In the meantime, and while we wait for the dawn of the 100% protein family structural coverage era, MR often remains a difficult problem, and especially so when the target has no homologues (or distant homologues only) available in the PDB. The contribution by Marco Marcia describes search models and MR for nucleic acids, which are one of the fastest expanding areas in structural biology, but face the difficulty that at the moment fewer than 5% of PDB entries contain nucleic acids. The papers on search ensembles from *ab initio* structure prediction in *Rosetta* (Frank di Maio), the extension of *AMPLE* to solution NMR structures (Daniel Rigden), and the use of normal mode perturbation and SCED score (Airlie McCoy) all expand the arsenal of search models for attempting MR structure solution in difficult cases.

The use of small and/or weakly homologous fragments as search models is also an important contemporary trend in MR, but of course the smaller and less structurally homologous the fragment, the smaller the signal and the more serious the difficulty in improving the initial MR phases. The papers by Randy Read, Andrea Thorn and Tom Terwilliger in this issue discuss small (down to one atom!) search fragments, means to assess the degree of structural homology of a search model, and post-MR phase improvements. Kevin Cowtan’s method of density modification, acting directly on the electron density obtained from the MR phases, without the need for an atomic model, was presented in its first implementation at the meeting, but not submitted for publication.

The future of course will bring crystals and electron microscopy (EM) and tomography images of larger and larger macromolecular assemblies: the contributions by Debora Makino, David Barford and Nicola Abrescia illustrate the use of MR in the structure solution of multi-subunit macromolecular complexes and viruses, based on X-ray and/or electron microscopy data. And given that technology shapes so much of present-day cutting-edge scientific research, the MR automated pipelines described by Gábor Bunkóczi and Chantal Abergel in their papers prove that MR software developers too are well placed and determined to profit from the wonders of current computing power.

Finally, we must not forget the CCP4 Study Weekends of 1985, 1992, 2001 and 2007, all devoted to MR: almost 30 years have passed since the first volume of CCP4 Study Weekend Proceedings on the topic, and six years since the most recent one. The work gathered in this issue builds upon and adds to the results of that past, and captures the progress made since. Giovanna Scapin’s article takes us from the early days of MR, through the developments in *AMoRe, X-PLOR* and *MOLREP*, and all the way to the contemporary likelihood-based methods in *Phaser*. Martin Noble’s introductory lecture took us through the fundamentals of molecular replacement, along with the metaphor that MR is a transplantation of phases from one structure into another. And if we needed one extra reminder of the power and importance of the method, Andrew Kruse’s paper describes the MR structure determination of the G protein-coupled receptor proteins, for whose discovery and biochemical characterization the Nobel Prize for Chemistry has been awarded as recently as 2012.

